# A Model for Determining Predictors of the MUAC in Acute Malnutrition in Ghana

**DOI:** 10.3390/ijerph18073792

**Published:** 2021-04-05

**Authors:** Smart Asomaning Sarpong, Abena Kyeraa Sarpong, Youngjo Lee

**Affiliations:** 1 Senior Research Fellow, Centre for Social Science Research, Kumasi Technical University, Kumasi 854, Ghana; 2 Biomedical Scientist, Department of Laboratory Technology, Kumasi Technical University, Kumasi 854, Ghana; sarpongab19@gmail.com; 3 Department of Statistics, Seoul National University, Seoul 08826, Korea; youngjo@snu.ac.kr

**Keywords:** malnutrition, MUAC, Ghana, generalized linear model

## Abstract

The issue of malnutrition is perhaps the most important public health determinant of global wellbeing. It is one of the main causes of improper mental and physical development as well as death of many children. The Mid Upper Arm Circumference (MUAC) rapid text setup is able to diagnose malnutrition due to the fact that the human arm contains subcutaneous fat and muscle mass. When proportional food intake increases or reduces, the corresponding increase or reduction in the subcutaneous fat and muscle mass leads to an increase or decrease in the MUAC. In this study, the researchers attempt to develop a model for determining the performance of MUAC in predicting Child malnutrition in Ghana. It focuses on the Joint Generalized Linear Model (Joint-GLM) instead of the traditional Generalized Linear Model (GLM). The analysis is based on primary data measured on children under six years, who were undergoing nutritional treatment at the Princess Marie Louise (PML) Children’s Hospital in the Ashiedu Keteke sub-metro area of Accra Metropolis. The study found that a precisely measured weight of a child, height, and albumen levels were positive determinants of the predicted MUAC value. The study also reveals that, of all the variables used in determining the MUAC outcome, the hemoglobin and total protein levels of a child would be the main causes of any variation between the exact nutritional status of a child and that suggested by the MUAC value. The final Joint-GLM suggests that, if there are occasions where the MUAC gave false results, it could be a result of an imbalance in the child’s hemoglobin and protein levels. If these two are within acceptable levels in a child, the MUAC is most likely to be consistent in predicting the child’s nutritional status accurately. This study therefore recommends the continued use of MUAC in diagnosis of child malnutrition but urges Ghana and countries in Sub-Saharan Africa to roll out an effective nutrition intervention plan targeting the poor and vulnerable suburbs so that the nutritional status of children under five years of age, who were the focus of the current study, may be improved.

## 1. Introduction

Malnutrition remains an important public health issue all over the world. It is seen by many stakeholders to be the main cause of improper mental and physical development of children. Studies have shown that whereas about a quarter of the children less than five years of age are stunted worldwide, about 9% of the children in Sub-Saharan Africa have moderate acute malnutrition with about 2% of them having severe acute malnutrition [1], and nearly a 100% of all such children who die live in developing countries [2]. Again, in Africa, mortality is associated with the severity of malnutrition with severe wasting having a mortality rate of 73–187 per every 1000 children in a year [3]. Ghana’s case is not so different from the worldwide and African situation. Here in Ghana, malnutrition is said to contribute to about half of all deaths of children less than five years with some 12,000 of them dying every year of underweight related ailments [4].

The reason for use of the Mid Upper Arm Circumference (MUAC) in the diagnosis of malnutrition lies in the fact that the human arm contains subcutaneous fat and muscle mass. When proportional food intake reduces, the corresponding reduction in the subcutaneous fat and muscle mass leads to a decrease in the MUAC. Many researchers have therefore continually recommended the use of this rather very simple-to-use and low-cost tool in screening for malnutrition among children less than five years of age ([5,6,7,8]).

Even though MUAC is used as a rapid indicator for screening for acute malnutrition in children, its cut-offs remain controversial among researchers and other stakeholders. In their recent study, Fiorentino et al., [9] tried to define gender and age-specific cut-offs to improve the sensitivity of MUAC. They argued that, MUAC cut-offs currently being used by the World Health Organization [10], is limited in its ability to identify the majority of children whose weight-for-height Z-scores are <−2 (moderately malnourished) or <−3 (severely malnourished). Their study found boys to have higher cut-offs than girls with the general optimal cut-off values increasing with age, hence the need for gender and age specific MUAC cut-offs [9].

The agreement between MUAC and weight-for-age also appears to vary within different groups of children [11]. It is therefore not clear which one of them may prove to be the most adequate predictor of clinically diagnosing malnutrition compared to that suggested by the MUAC. The objective of this study is to develop a model for determining the performance of MUAC in predicting Child malnutrition in Ghana using variables, such the child’s age, gender, height, weight, albumen level, total protein, as well as their interaction terms. In line with the global quest to develop statistical models with the highest accuracy and precision, we seek to compare results from the traditional generalized linear model to that of the joint generalized model. The final model of this study shall be the one with extensive improvement over the other, which is also able to identify variables that are likely to account for an increase or decrease in the precision of the MUAC value.

The use of MUAC to identify children for admission into nutritional treatment has been well explored [12,13] as well as criticisms in respect of its setbacks [14,15]. This study takes notice of the potential risk of misclassification of a child’s nutritional status and the possible deaths that may occur should a malnourished child be wrongly classified as well-nourished. The study therefore combines well-explored determinants of malnutrition, such as weight, age, height of a child (and their interaction terms; W*H, H*A, W*A), as well as some clinical determinants (albumen, protein and hemoglobin) in the modeling with the aim of selecting the key determinants.

This study is relevant in both design and purpose. In design, the choice of statistical modeling in the study of an important public health issue shall enable us to identify the key variables relevant for the adequate description of a given subpopulation by the reliance upon some mathematical expressions representing their distributions. Most important to this study is our interest to identify possible sources of discrepancy in the MUAC’s ability to predict malnutrition in children under five years of age. The study therefore explores the Joint-GLM approach to elicit the two main objectives of this study. In purpose, this study adds to the ongoing discussion on malnutrition in children under five years of age with the aim of contributing to the knowledge on the possible occasions where the MUAC may give false results.

## 2. Method

### 2.1. Data Source and Measurement

Data for this study were obtained from a cross-sectional survey conducted between December 2018 and May 2019. The study setting was the pediatric unit of the Princess Marie Louise children’s Hospital (PMLCH) in the Ashiedu Keteke Sub-Metro of the Accra metropolitan assembly. In all, a sample of 163 malnourished children were used for our analysis and reporting. Variables measured include MUAC as the dependent variable, with a child’s age, height, weight, hemoglobin level, total protein, and albumen as independent variables. Data cleaning and editing was done daily.

Two trained personnel assisted in the process of data collection; one collecting data on MUAC, age, weight, and height, while the other assisted in recording clinical data (albumen, hemoglobin and protein) that were made available to us by the laboratory department of the hospital. Recumbent length was measured for children less than 24 months while standing height was measured for children 24 months to five years. The Schorrboard (Schorr Production, MD, USA) was used in measuring length and recordings were made to the nearest 0.1 centimeter (cm). In the measurement of weight of a child, the Seca 874 scale (Seca Gmbh & Co. KG, Humburg, Germany) was used. Weights were recorded to the nearest 0.1 kilogram. MUAC was measured to the nearest 0.1 millimeter (mm) using the Johns Hopkins University MUAC tape.

### 2.2. Ethical Approval

Ethical approval was sought and obtained from the PML children’s hospital. Informed consent was sought from the mothers and caregivers of each participant. The right of participants to decline participation was strictly emphasized and respected throughout the study data collection. Data have been and would continually be treated with absolute confidentiality.

### 2.3. Data Analysis

The R-console statistical software was used in analyzing the data and generating all diagnostic plots. Descriptive analysis was used first to summarize the data, then correlation analysis was done to explore the relationship between the variables under study.

### 2.4. Statistical Methods for MUAC Model

In theory, the traditional generalized linear model (GLM) is obtained from classical linear models by two extensions, one to the random part and another one to the systematic part [16]. By these extensions therefore, the random elements are now allowed to belong to a one-parameter exponential family including the normal distribution. Since its inception, GLMs have been used as technique for analyzing data in various types of responses, continuous quantities, counts, proportions, and positive quantities. They allow the regression (or fixed effect) model only for the mean of independent responses [17]. Model checking is usually based on examination of the model’s diagnostic residuals, similar to the linear model case, except that in the case of GLM’s, standardization of residuals is required and is a little difficult to perform.

In practice, even though the GLM is widely noted for its good performance in modeling, some natural discrepancies arise amongst the data and the fitted values generated. Observations that have large discrepancies on the *y*-axis are known as outliers. Discrepancies amongst the data and the fitted values generated by GLMs fall into two main classes; isolated or systematic [18]. When few observations have large residuals, isolated discrepancies are seen. Such residuals can occur if the observations are wrong. An example is when 19 is recorded as 91. Data-driven robust methods are sometimes used in studies to handle such cases. However, such robust methods are unable to identify the causes of the discrepancies.

An alternative is to model isolated discrepancies as being caused by variation in the dispersion, and to seek covariates that may account for them. This technique of joint modeling of the mean and dispersion known as Joint Generalized Linear Models [18] makes such exploration straightforward. Furthermore, if a covariate can be found to be the cause of any discrepancies, then we obtain a model-based solution which can be altered in the future by policy makers in the field it is applied. We therefore compare the results from the traditional generalized linear models to those of the joint generalized model to justify that Joint-GLMs are appropriate in determining predictors of the MUAC value.

### 2.5. Models Fitted

Since the MUAC value observed $Y_{i}$ (for i=1,2,3,…,163) is the dependent/response variable with age, weight, height, hemoglobin, protein, and albumen as independent variables, the following models were fitted for the traditional as well as the Joint -GLMs.

#### 2.5.1. Generalized Linear Models

The generalized linear model used in this study is a Gaussian model consisting of three components:

A random component specifying the conditional distribution of the response variable $Y_{i}$ given the explanatory variables.

A linear function of the regressors called the linear predictor,

(1) $$\eta_{i}=\beta_{0}+X_{ij}\beta_{j}+\cdots +X_{ik}\beta_{k}=x_{ij}^{\prime }\beta$$

where *i* = 1, 2, 3, …, n [number of participants], *j* = 1, 2, 3, …, 6 [number of independent variables] on which the expected value $\mu_{i}$ of $Y_{i}$ depends.

An invertible $\mathrm{link}\;\mathrm{function}\;g\left(\mu_{i}\right)=\eta_{i}$, which transforms the expectation of the response to the linear predictor. The inverse of the link function is sometimes called the mean function: $g^{-1}\left(\eta_{i}\right)=\mu_{i}$.

#### 2.5.2. Joint Generalized Linear Models

The method used in this paper follows the Joint-GLMs of Lee and Nelder [17]. By their method, we have two interlinked models; one for the mean and another one for the dispersion based on same observed data y and the deviance d:

(2) $$E\left(y_{i}\right)=\;\mu_{i},\;\;\eta_{i}\;=\;g\left(\mu_{i}\right)\;=\;x_{i}^{t}\beta ,\;\;var\left(y_{i}\right)\;=\;\phi_{i}V\left(\mu_{i}\right)$$

(3) $$E\left(d_{i}\right)=\;\phi_{i},\;\;\xi_{i}\;=h\left(\phi_{i}\right)\;=\;g_{i}^{t}\beta ,\;\;var\left(d_{i}\right)\;=\;2\phi_{i}^{2}$$

where $\xi_{i}$ is the link function of the dispersion model, $g_{i}$ is the model matrix in the dispersion model, which is a GLM with a gamma variance function and $x_{i}$ is the model matrix in the mean ($\mu_{i}$) model. This implies that the algorithm for fitting these models can be reduced to the fitting of a two-dimensional set of generalized linear models; one dimension being the mean and the other being the dispersion, so that no special code is required for the estimation of the dispersion components. In the Joint-GLM, the dispersion parameters are no longer considered to be constants, as it is in the case for the traditional GLM, but can vary with the mean parameters. This means that the dispersion values are required in the iterative weighted least squares (IWLS) algorithm for estimating the regression parameters. These values have a direct effect on the estimates of the regression parameters. The deviance components d become the response variable for the dispersion GLM.

### 2.6. Model Fittness and Elimination of Statistical Bias

Model selection was done by computing and assessing three very useful criteria in statistical modeling; the Akaike Information Criterion (AIC), the conditional-AIC (cAIC), and the Bayesian Information Criterion (BIC). These criteria enhance the exploratory power of statistical models while penalizing model complexities. As a rule, when the three criteria disagree on the best-fit model by generating varying values, we choose the model with the lowest AIC. This is done to guard against false negative models. In this study, therefore, the best model shall be adjudged to be that with the lowest value for the Akaike information criterion (AIC), the Bayesian Information criterion (BIC), and the conditional Akaike information criterion (cAIC). The data-driven statistical bias was minimized by the rigorous selection of variables selected only upon securing a minimum *p*-value <0.2 in a preliminary exploratory backward elimination procedure. Correlations among independent variables (multi-collinearity) were examined and we can report that independent variables used in this analysis are not collinear.

## 3. Results and Discussion

This study measured height (length) and weight of children as well as the MUAC and other biomedical indicators used in malnutrition determination [19]. The descriptive result is presented in Table 1. From that table, the age of the children studied ranges from half a year to five years with a mean of 1.6 years. The minimum height was 20 cm with the maximum height recorded as 160 cm and a mean height of 78.5 cm. The weights of these children ranged from 1.75 kg to 20 kg (mean = 9.4 kg), with hemoglobin levels found to be within 2.1 to 19.4 milMol/L (mean = 10.2). The minimum total protein was found to be 50 and the maximum was 84 with a mean of 65.5. The study also found albumen levels ranging from 22 to 48 milMol/L. Finally, the minimum and maximum MUAC were found to be 7.4 to 17.5 cm, respectively, with a mean value of 13.1 cm.

Table 2 presents correlation values between the dependent variable and their explanatory valuable. A child’s age is found to have a very strong correlation with the weight and height which are 0.799 and 0.709, respectively, but had a rather moderate correlation with the MUAC and the total protein (0.43 and 0.22, respectively). There exists however a rather weak correlation (0.155) between the age of a child and albumen levels.

**Figure 1:**
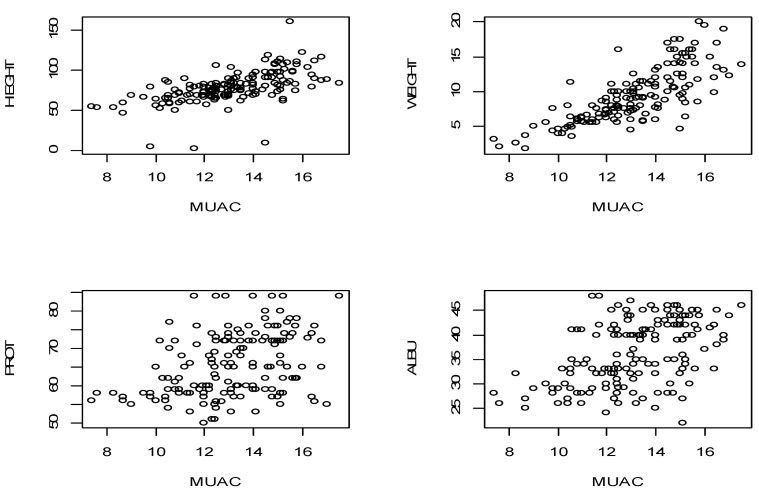
Scatter plots for selected parameters versus Mid Upper Arm Circumference (MUAC).

Similarly, the weight of a child is highly correlated with the MUAC and the height of a child, the values are 0.768 and 0.773, respectively. Our dependent variable MUAC, however, showed a moderate correlation with all the dependent variables except for the weight of a child. Similarly, the weight of a child is highly correlated with the MUAC and the height of a child, the values are 0.768 and 0.773, respectively. Our dependent variable MUAC, however, showed a moderate correlation with all the dependent variables except for the weight of a child. Figure 1 shows scatter plots of the inter-correlations between the variables.

### 3.1. Modelling

From the parameter estimates (Table 3), the fitted traditional Gaussian GLM selected the following linear determinants as those that significantly influence the accuracy of the MUAC. They include age, weight, and height of a child as the fixed terms, as well as their interactions weight * height and height*age after a t-test (approximated to Z due to the large sample size) on individual parameters. On the other hand, hemoglobin levels, age*weight, albumen and protein levels were not found to be significant. In reporting interaction terms, where the main variable is only significant upon interaction, the otherwise insignificant variable is deemed to be significant. In this model, however, the variable whose interactions were significant in predicting the MUAC were themselves significant.

**Figure 2:**
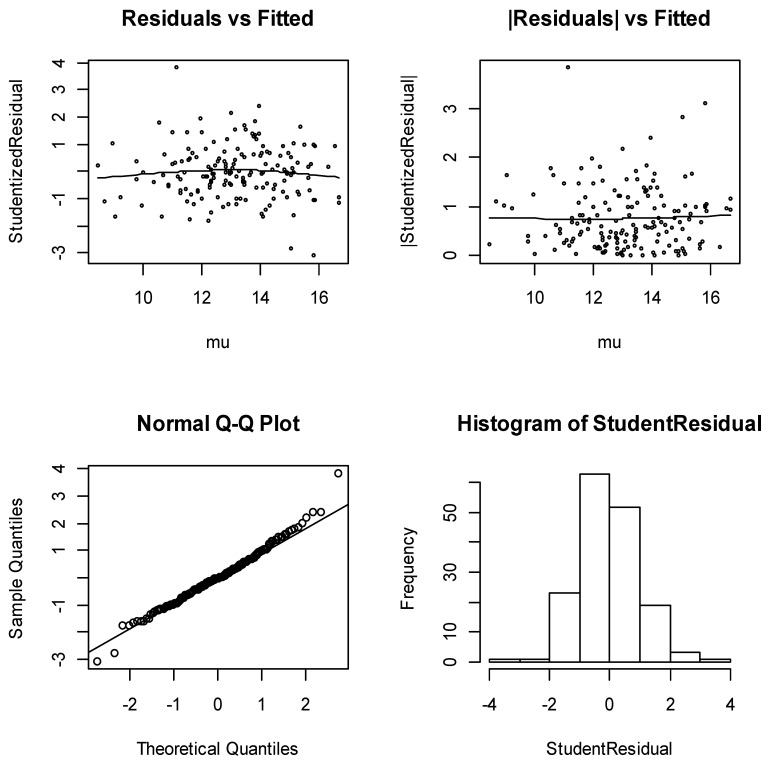
Diagnostic plots for Gaussian GLM.

Before applying the distributional results for inference, it is always necessary to check that the model meets its assumptions well enough to be sure that the results are likely to be valid. Figure 2 shows the model-checking plots for the traditional Gaussian model. From Figure 2, the diagnostic plots have several satisfactory features. The running mean in the plot of residuals against fitted values shows no form of a marked trend, and the plots of the absolute residuals have a relatively stable slope. The normal plots show no discrepancy. In addition, the histogram of the residuals is almost symmetric to the left. These are very good indications of an appropriate model.

### 3.2. Joint-Generalized Linear Models

**Figure 3:**
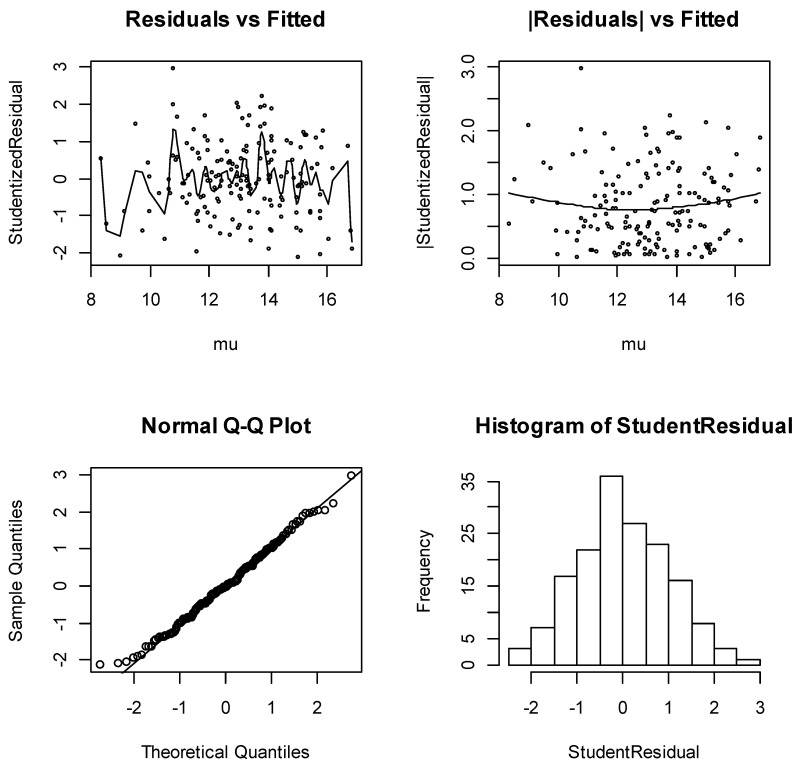
Diagnostic plots for joint Gaussian GLM.

Similarly, Figure 3 shows the model-checking plots for the Joint Gaussian model. From that figure also, the diagnostic plots have satisfactory features. The running mean in the plot of the residuals against the fitted values shows no form of a marked trend, and the plots of absolute residuals have a relatively stable slope. The normal plots show no discrepancy and the histogram of the residuals is almost symmetric to the left. These are very good indications of an appropriate model. Table 4 reveals that even though the traditional GLM was a satisfactory mean model, modeling both mean and dispersion (Joint-GLM) improves the quality of the model diagnostics significantly.

### 3.3. Discussion

On all the three bases for best model selection (see Table 5) the Akaike information criterion (AIC), the Bayesian Information criterion (BIC), and the conditional Akaike information criterion (cAIC), the Joint-GLM performs far better compared to their GLM counterpart. The primary condition for decision is that the best model is the one with the least criteria values. This study therefore settles on the Joint GLM for inference. The fixed terms in Table 3; age, weight, height, and albumen, were confirmed to significantly determine the accuracy of the MUAC whereas protein and hemoglobin were not. If we therefore wish to rely on the MUAC for predicting acute malnutrition in children under five, then we have to do all we can to ensure that the age, weight, height and albumen parameters are determined correctly. The selection of albumen by our model based on the data analyzed is supported by Chowdhury et al. [14].

Age is found to be highly determinant for the accuracy of MUAC (coefficient = −2.477) but it does so inversely. This implies that, as you grow, the ability of the MUAC to accurately predict acute malnutrition reduces. Fiorentino et al. [9] recommended the need for gender- and age-specific MUAC cut-offs. The findings of this study also support the call for an age-specific MUAC cut-off. The WHO is hereby impressed upon to pay more attention to age-specific cut-offs. All other fixed terms age, weight, height, and albumen levels of a child are found to significantly influence the accuracy of the MUAC directly.

From the dispersion model in Table 6, however, a very important piece of information is revealed. It is observed that relying on the mean Joint-GLM in determining the MUAC accuracy, we record a possible dispersion of −3.686 (intercept of the dispersion model). This variation/dispersion may serve as a good confirmation of the report by Grellety and Golden [20] to the effect that some children aged 6–59 months are falsely diagnosed as malnourished when the weight-for-height (WH) and MUAC are used. Another important piece of information from the dispersion model of the Joint-GLM is its ability to reveal the contribution of each determinant to increasing or decreasing the dispersion. For instance, we observe that of the two variables hemoglobin and protein levels, which introduce significant discrepancies into the accuracy of the MUAC, a less balanced hemoglobin level of a child will lead to an increase in the discrepancy regarding the predicted MUAC whereas that of the protein levels will reduce any such discrepancy.

In the anthropometric study of Grellety and Golden [20] in some 47 countries, the discrepancies in the two indices (weight-for-height (WH) and MUAC) were found to vary from country to country with the majority diagnosed as malnourished when both criteria are used strictly. So, in many instances, MUAC and WH have been used as complementary indicators rather than alternative determinants in examining malnutrition. The significance of this current study model is that, once a variable is found to account for a discrepancy, then we achieve a model-based solution to the question of which variables should be re-measured, modified, or even ignored by policy makers regarding the nature of discrepancy they introduce.

There are several strengths of this study. Data for the study came from a population-based sample hence providing a useful representation of the Ashiedu-Keteke sub-metropolitan area as whole. Misclassification was minimized significantly with the use of standardized data collection tools and triplicate measurements of anthropometric indicators. Again, whereas many models used in Child malnutrition studies are unable to establish a causal relationship between the observed dependent and independent variables, our Joint-GLM is able to establish such a relationship demonstrably well, thereby improving the quality of the model. Our model is able to identify sources of discrepancy in reaching accuracy, making it possible for policy makers to determine the fate of such variables in the future. However, one notable limitation of this study includes the sample size, which was admittedly small to examine moderate to severe acute malnutrition. Some previous studies [21,22] have opined that large samples should be used in MUAC models. That notwithstanding, the findings of this study are generalizable to populations with shared characteristics making it very useful for policy formulations. Another likely limitation of this study would be the generalizability of the results beyond the setting of this study. The general pattern of malnutrition, infectious diseases, and child mortality in Ashiedu-Keteke is different to the other more elite parts of the Accra metropolitan area. In addition, despite the use of a sufficient set of predictor variables in our analysis, the many more sets of covariates unmeasured and unconsidered makes it imperative to mention the possibility of bias due to residual confounding.

### 3.4. Policy Implications

An imbalance hemoglobin level is an indication of an iron-deficiency anemia usually resulting from low consumption of fish, meat, vegetables, and also due to general low intake of proteins. For the two determinants (hemoglobin and protein) to be accounting for this discrepancy, policy makers may wish to address the possible food insecurity likely to be the main underlying cause of malnutrition for households from where these children are coming. Intervention strategies aimed at improving knowledge of caregivers on the subject of nutrition as well as food supply to these caregivers from poor socio-economic homes. The time for an effective nutrition intervention plan targeting the poor and vulnerable suburbs in Ghana has been long overdue.

In addition, this study identifies hemoglobin and protein as the key clinical factors that might trigger the all useful MUAC to be unreliable. These two clinical factors will assist in the proper allocation of health resources aimed at improving child nutrition at both the individual and community levels; most especially in poor socio-economic homes.

On the basis of the study outcomes, the ministry of health and the government of Ghana need to critically consider formulating and implementing community-based interventions to improve child health. At the individual level, caregivers especially mothers should be adequately educated in basic nutrition and the need to frequently visit health facilities to be sure of the child’s nutritional status. In addition, mother–child programs should be made readily accessible to women in poor communities across Ghana by our health care system. It is our hope that these interventions, if considered by Ghana and countries in Sub-Saharan Africa, may go a long way to improve the nutritional status of children under the age of five years, who were the focus of the current study.

## 4. Conclusions

This study selects seven key determinants of the accuracy of MUAC in the determination of malnutrition of children under the age of five years. They include age, height, weight, and their interactions (H*A, W*H, W*A), albumen level of a child. More importantly, the model suggests that, if there are occasions where the MUAC gave false results, it could be a result of an imbalance in the child’s hemoglobin and protein levels. If these two are within acceptable levels in a child, the MUAC is most likely to be consistent in predicting child nutritional status accurately.

We again conclude that even though the traditional GLM is still a satisfactory mean model, modeling the mean and dispersion (Joint-GLM) improves the quality of the models significantly and also provides two very important pieces of information; the value of discrepancy, and the actual variables causing it. Once any variable is found to account for a discrepancy, policy makers can then decide what should be done in respect of the particular disturbing variable. They may decide to re-measure, modify, or even ignore depending on the nature of the discrepancy they introduce.

## Figures and Tables

**Table 1:** Descriptive statistics.

Item	Minimum	Maximum	Mean	Median	Std. Deviation	N
Child’s age (years)	0.13	5	1.6334	1.0000	1.18655	163
height of child (cm)	20	160	78.5706	78.0000	19.38403	163
weight of child (kg)	1.75	20	9.3894	8.8000	3.90184	163
Hemoglobin	2.1	19.4	10.2031	10.6000	1.84967	163
Total protein	50	84	65.5853	65.0000	8.43823	163
Albumen	22	48	36.226	35.000	6.5421	163
M.U.A.C.	7.4	17.5	13.1200	13.1000	1.98962	163

**Table 2:** Correlations between variables.

Items	Age	Weight	M.U.A.C.	Haem	Protein	Albumen	Height
Child age	1						
weight of child	0.799 **	1					
M.U.A.C.	0.430 **	0.768 **	1				
Hemoglobin	0.173 *	0.250 **	0.303 **	1			
Total protein	0.222 **	0.337 **	0.381 **	0.295 **	1		
Albumen	0.155 *	0.373 **	0.477 **	0.300 **	0.658 **	1	
height of child	0.709 **	0.773 **	0.589 **	0.276 **	0.302 **	0.268 **	1

**. significant at the 0.01 level (2-tailed). * significant at the 0.05 level (2-tailed).

**Table 3:** Model estimates for Gaussian Generalized Linear Model (GLM).

Items	Estimate	Std. Error	t-Value
(Intercept)	3.297646	1.112371	2.9645 ***
Age	−2.44933	0.706229	−3.4682 ***
Weight	1.214475	0.182097	6.6694 ***
Height	0.046291	0.016303	2.8394 ***
Hemoglobin	0.061822	0.047596	1.2989
Protein	0.010197	0.013159	0.7749
Albumen	0.024162	0.017901	1.3497
Weight: Height	−0.007833	0.002123	−3.6900 ***
Age: Weight	−0.024477	0.028805	−0.8498
Age: Height	0.022875	0.008388	2.7271 ***
*** = sig at *p* < 0.00			
Likelihood Function Values and Condition AIC			
-2ML (-2 h)		464.2776	
-2RL (-2 p_beta (h))		527.8103	
cAIC		484.2776	

**Table 4:** Model Estimates for Gaussian Joint-GLM.

Items	Estimate	Std. Error	t-Value
(Intercept)	3.514933	0.699909	5.022 ***
Age	−2.161377	0.522314	−4.138 ***
Weight	1.194023	0.127182	9.388 ***
Height	0.028779	0.008209	3.506 ***
Hemoglobin	0.033497	0.030733	1.090
Protein	0.016998	0.011937	1.424
Albumen	0.032157	0.015628	2.058 **
Weight: Height	−0.006908	0.001439	−4.800 ***
Age: Weight	−0.051909	0.021880	−2.372 **
Age: Height	0.024228	0.006006	4.034 ***

** = sig at *p* < 0.01, *** = sig at *p* < 0.00.

**Table 5:** Model criteria for Gaussian GLM and Gaussian Joint-GLM.

Model Criteria	Gaussian GLM	Gaussian Joint-GLM
-2ML (-2 h)	464.2776	438.4326
-2RL (-2 p_beta (h))	527.8103	506.8090
cAIC	484.2776	458.4326

**Table 6:** Dispersion model estimates for Gaussian Joint-GLM.

Items	Estimate	Std. Error	t-Value
(Intercept)	−3.686e+00	1.721319	−2.1414 **
Age	−4.625e−01	0.986622	−0.4687
Weight	1.549e−01	0.256311	0.6043
Height	4.431e−02	0.027394	1.6175
Hemoglobin	2.719e−01	0.065510	4.1505 ***
Protein	−4.087e−02	0.017389	−2.3503 **
Albumen	3.496e−02	0.023711	1.4744
Weight: Height	−3.353e−03	0.003065	−1.0939
Age: Weight	3.820e−02	0.044118	0.8656
Age: Height	1.095e−05	0.012225	0.0009
** = sig at *p* < 0.01, *** = sig at *p* < 0.00			
Likelihood Function Values and Condition AIC			
-2ML (-2 h)		438.4326	
-2RL (-2 p_beta (h))		506.8090	
cAIC		458.4326	

## Data Availability

The data presented in this study are available on request from the corresponding author. The data are not publicly available due to ethical reasons.
